# Residual Pulmonary Hypertension More than 20 Years after Repair of Shunt Lesions

**DOI:** 10.3390/medicina56060297

**Published:** 2020-06-16

**Authors:** Dovilė Jančauskaitė, Virginija Rudienė, Gabrielius Jakutis, Laurie W Geenen, Jolien W Roos-Hesselink, Lina Gumbienė

**Affiliations:** 1 Centre of Cardiology and Angiology, Vilnius University, LT 08661 Vilnius, Lithuania; lina.gumbiene@santa.lt; 2 Faculty of Medicine, Vilnius University, LT 03101 Vilnius, Lithuania; vr.rudiene@gmail.com (V.R.); jakutis.gabrielius@gmail.com (G.J.); 3 Department of Cardiology, Erasmus MC University Medical Center Rotterdam, 3000 CA Rotterdam, The Netherlands; l.geenen@erasmusmc.nl (L.WG.); j.roos@erasmusmc.nl (J.WR.-H.); 4 Centre of Heart and Chest Surgery, Vilnius University, LT 08661 Vilnius, Lithuania

**Keywords:** pulmonary hypertension, congenital heart disease, surgery, right heart catheterisation

## Abstract

*Background and Objectives:* After successful surgical repair of a congenital shunt lesion, pulmonary hypertension (PH) often disappears. However, PH can persist long-term after the closure. This study aimed to assess the prevalence of PH long-term after surgical repair of congenital heart disease (CHD), and to evaluate the outcomes and preoperative factors related to residual PH. *Materials and Methods:* In this retrospective cohort study, we reviewed patients who underwent right heart catheterisation in Vilnius University Hospital Santaros Klinikos during the period of 1985–2007. Among 4118 right heart catheterisations performed, 160 patients underwent congenital systemic-to-pulmonary shunt repair at a young age (<18 years) and had pre-operative PH. Half of the patients were foreigners whose follow-up data were unavailable. Eventually, 88 patients with available follow-up data were included in this study. *Results:* The median age at diagnosis of CHD with PH was 0.8 (0.6–3.0) and 1.1 (0.6–3.9) years at surgery (50% females). Residual PH was assessed 9.5 years after surgery and observed in 30.7% (*n* = 27) of the patients. It was associated with having more than one shunt (44.4% (*n* = 12), *p* = 0.016) and higher median pulmonary vascular resistance (3.4 (2.5–6.5) vs. 2.2 (1.0–3.7), *p* = 0.035) at baseline. After a median follow-up of 21 (15–24) years, 9.1% of the patients were deceased. Kaplan–Meier survival analysis revealed significantly higher mortality in the residual PH group (*p* = 0.035). *Conclusions:* Residual PH affects a significant proportion of patients after surgical repair of a shunt lesion and is associated with worse long-term outcome.

## 1. Introduction

Pulmonary hypertension (PH) is one of the most severe complications in congenital systemic-to-pulmonary shunts [1]. Despite early surgical repair, pulmonary arterial hypertension (PAH) is found in 2–6% of children and 5–13% of adult patients with congenital heart defects (CHD) [2,3,4]. PAH after surgically repaired CHD is associated with worse outcomes compared to other aetiologies of PAH [5]. Despite recent advances in management of PH and published PH treatment guidelines, a “grey zone” in deciding whether to correct still exists [1]. Without CHD repair, systemic-to-pulmonary shunting increases pulmonary blood flow, pressure in the pulmonary arteries, and vascular resistance, and leads to reversal of the systemic-to-pulmonary shunt, cyanosis, hypoxemia, and severe inoperable PAH due to CHD, known as Eisenmenger syndrome [1,6]. Therefore, accurate timing of the operation is crucial and the quest for precise operability assessment between reversible and irreversible PH is ongoing [7]. Early diagnosis and surgery of CHD results in more patients reaching adulthood, hence an expanding population of adults with CHD [8,9]. Despite achievements in early management, many patients suffer from lifelong complications, such as residual lesions warranting new interventions, arrhythmias, heart failure, thromboembolism, and PH [10,11].

The aim of our study was to assess the prevalence of PH long-term after surgical repair of congenital systemic-to-pulmonary shunts and to evaluate the outcomes and preoperative factors related to residual PH.

## 2. Materials and Methods

### 2.1. Study Design

**Figure 1:**
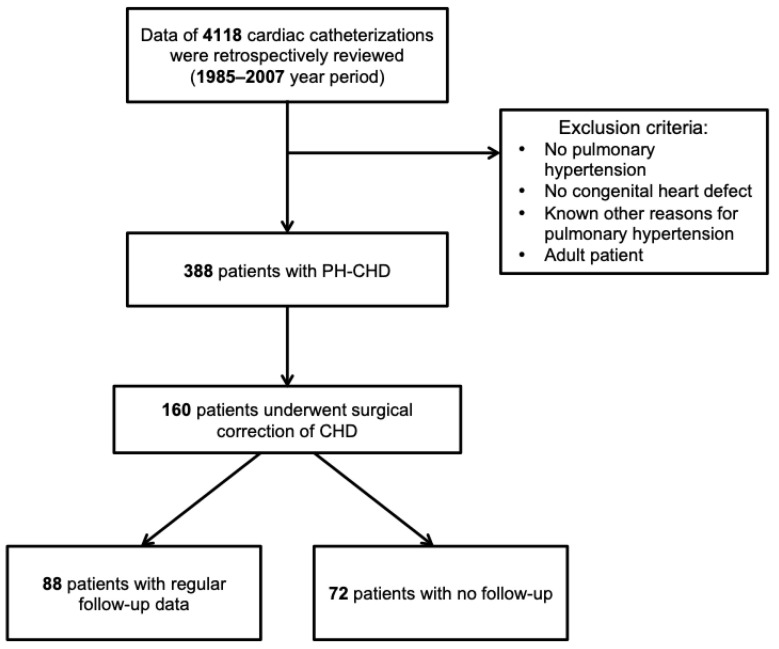
Flowchart of the patient selection process. PH—pulmonary hypertension; CHD—congenital heart disease.

In this retrospective cohort study, we reviewed patients who underwent right heart catheterisation in Vilnius University Hospital Santaros Klinikos during the time period of 1985–2007. We identified all patients who underwent congenital systemic-to-pulmonary shunt repair at a young age (<18 years) and had pre-operative PH. The study was conducted in accordance with the Lithuania national guidelines on human experimentation and the Declaration of Helsinki, and has been approved by the Academic Ethics Commission of the Faculty of Medicine of Vilnius University (no. 158200-15-822-33, approved 08.12.2015). PH was defined as mean pulmonary artery pressure ≥25 mmHg at rest as determined during the right heart catheterisation [1,12]. The procedure was performed under general anaesthesia, and oximetry was measured after stopping oxygen supply for 15 min. The direct Fick method was used for cardiac output measurements. If there were no data on mean pulmonary artery pressure, we included patients according to systolic pulmonary artery and aortic pressures (systolic pulmonary artery pressure >1/2 systolic aortic pressure). Study inclusion and exclusion criteria are presented in Figure 1. The date of the PH–CHD diagnosis was defined as the date of right heart catheterisation. Incidence of airway obstruction was not analysed during this study. For long-term outcome evaluation, we only included patients who underwent surgical repair of CHD and had regular follow-up data until 2018 January, as obtained from the hospital’s electronic patients’ records system. Patients who came for the surgical procedure from other countries and who had no follow-up data in our hospital system were excluded from the analysis. Assessment of residual PH after surgical CHD repair was determined either on the basis of right heart catheterisation when available or on echocardiographic data following the guidelines: high echocardiographic probability of PH according to peak tricuspid regurgitation velocity and presence of other echocardiographic signs of PH [1]. Survival status was determined on the basis of national health insurance data in the hospital electronic patients’ records system. Complications were assessed according to the last visit to the healthcare specialist. Note that one patient could have had more than one complication. The group of patients with regular follow-up data was compared with those without follow-ups. These patients were from another country and therefore lost to follow-up.

For the follow-up group, we stratified patients into two subgroups: patients with residual PH and without residual PH (non-residual PH group). The severity of PH was distributed according to the ratio of pulmonary artery and aortic pressures as follows: mild PH—pulmonary artery pressure <1/3 of aortic pressure; moderate PH—pulmonary artery pressure >1/3 and <2/3 of aortic pressure; severe PH—pulmonary artery pressure >2/3 of aortic pressure. According to the anatomical–pathophysiological classification, we divided patients into three groups: simple shunts (atrial septal defects (ASD), anomalous pulmonary venous drainage, ventricular septal defect (VSD), and patent ductus arteriosus (PDA)), combined (combination of simple shunts), and complex (complete atrioventricular septal defect, truncus arteriosus, transposition of the great arteries and tetralogy of Fallot) defects [13].

### 2.2. Statistical Analysis

Data were analysed using SPSS 24.0 (IBM, Armonk, New York, United States) statistical package. Du Bois formula was used to calculate the body surface area. Normality was assessed by one sample Kolmogorov–Smirnov test. On the basis of normality, numerical data is shown as means with standard deviations or as medians with interquartile ranges. *t*-test was used for analysis of normally distributed numerical variables between residual and non-residual PH patient groups. Mann–Whitney *U* test was performed for marginally skewed variables. Logistic regression was used to measure the association between the preoperative factors and residual PH and was only performed for variables which had <20% missing data. To compare the differences between types of systemic-to-pulmonary shunt, we used a one-way ANOVA test for variables with non-equal variances according to the Levene test, and the Kruskal–Wallis test was performed for non-normal distributed data.

Categorical data are expressed as numbers and percentages. Categorical preoperative factors within the groups were compared using chi-squared or Fisher’s exact test.

Survival was assessed with Kaplan–Meier analysis (from the first shunt repair until January 2018 or the last known date of follow-up). A log-rank test was performed to compare the survival distributions between residual and non-residual PH groups. For all tests, we considered a *p*-value less than 0.05 to be statistically significant.

## 3. Results

### 3.1. Baseline Characteristics and Haemodynamics

Of the total 4118 cardiac catheterisations, 388 patients were identified to have had CHD with PH and 160 underwent surgical repair of the shunt. Continuous follow-up data were available for 88 patients (Figure 1). A total of 72 patients had missing follow-up data due to moving or returning to other regions or countries.

Patients with and without follow-up were comparable on baseline characteristics. There were no statistically significant differences between these groups for variables including sex, mean age at surgery, height, weight, and body mass index, however, non-follow-up patients were diagnosed at a later age and had more combined and complex systemic-to-pulmonary shunts (see Table S1).

Baseline characteristics and haemodynamics according to shunt type and residual PH are presented in Table 1 and Table 2. The median age at diagnosis was 0.84 (0.61–3.03) years. The median time between PH diagnosis and shunt closure was 8 (5–69) days and median age at surgery was 1.07 (0.64–3.92) years. For 13.6% (*n* = 12) of patients, surgical defect repair was performed in the first 6 months of their life.

Simple systemic-to-pulmonary shunts were present in 40.9% (*n* = 36) of patients, among which a ventricular septal defect (29.5% (*n* = 26)) was the most common diagnosis. Less than one-third (27.3% (*n* = 24)) had combined simple shunts with the following combinations: ASD and VSD in nine patients, PDA and ASD in one patient, PDA and VSD in five patients, ASD and aortic coarctation (AoCo) in one patient, ASD and VSD and PDA in five patients, ASD and VSD and AoCo in one patient, and VSD and PDA and AoCo in two patients. About one-third (31.8% (*n* = 28)) of patients had a complex shunt, predominantly with a complete atrioventricular septal defect (15.9% (*n* = 14)) (Table 1). A genetic syndrome was diagnosed in 23 individuals (26.1%), with trisomy 21 being the most prevalent (69.6% (*n* = 16)). Patients with a combined shunt were diagnosed and corrected earlier than individuals with complex and simple shunts (0.65, 0.82, and 1.74 years, *p* = 0.012, 0.74, 0.99, respectively, and 2.21 years, *p* = 0.006). There was no significant statistical difference in terms of pulmonary artery pressures and severity of PH between the groups distributed by shunt type, however, diastolic and mean aortic pressures were the lowest in the complex shunt type group (*p* = 0.008 and *p* = 0.011, respectively).

### 3.2. Factors Related to Residual Pulmonary Hypertension

Assessment of residual PH was performed after a median of 9.5 (5.0–12.75) years after surgery, and 30.7% of patients had persistent PH after surgical repair of CHD (*n* = 27). Right heart catheterisation was performed in 12.5% (*n* = 11) of patients for the assessment of residual PH, and echocardiography was used for the majority of patients. Most of the patients with persistent PH had a combined shunt (44.4% (*n* = 12), *p* = 0.016) (Table 2). No significant age, sex, height, weight, and haemodynamics (right heart catheterisation)-related differences were observed between residual and non-residual PH groups, except for a higher median pulmonary vascular resistance in the residual PH group (3.4 (2.5–6.5) vs. 2.2 (1.0–3.7), *p* = 0.035). Male sex, age at diagnosis, and surgery were not significantly related to non-residual PH (Table 3).

### 3.3. Survival Analysis and Complications

**Figure 2:**
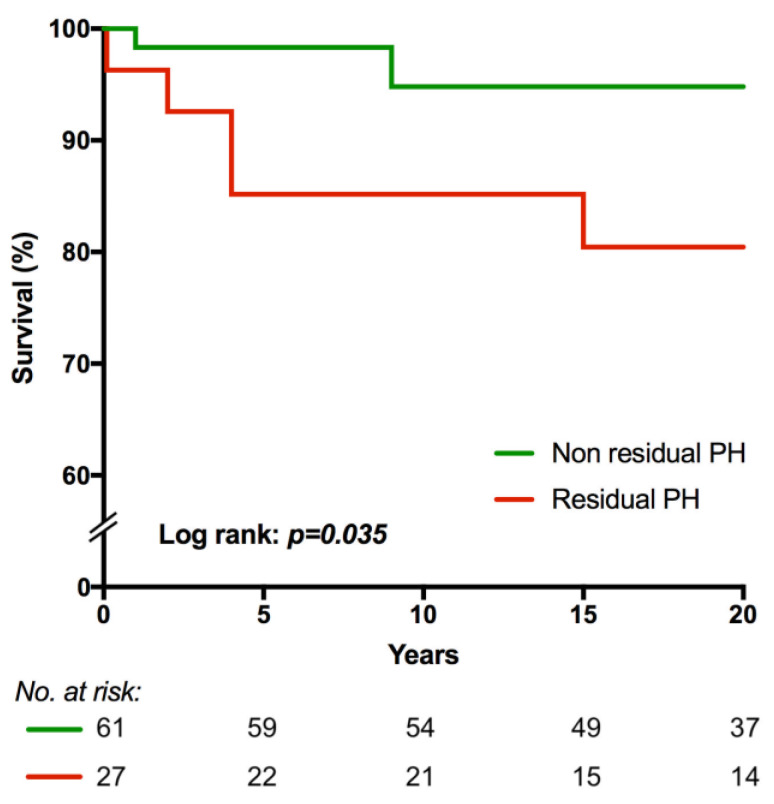
Kaplan–Meier survival analysis. PH—pulmonary hypertension.

After a median follow-up period of 21 (15–24) years, mortality rate was 9.1% (*n* = 8). The leading cause of death was heart failure (75% (*n* = 6)). Mortality rate was higher in patients with residual PH compared to patients without PH (5 (18.5%) vs. 3 (4.9%), respectively; *p* = 0.054) (Table 1). In the residual PH group, the underlying heart disease in patients with lethal outcome were VSD in one patient, complete atrioventricular septal defect (CAVSD) in one patient, combined defects in two patients, and complex defects in one patient; in the non-residual PH group: VSD in one patient, and complex in two patients. Kaplan–Meier survival analysis showed a significant difference in survival between these two groups (*p* = 0.035) (Figure 2).

The majority of patients were in NYHA (New York Heart Association heart failure classification) class 2 and 3 at the last follow-up (Figures S1 and S2). Residual defects (due to partial patch dehiscence or non-complete closure) were observed in 27.3% of patients, and re-operation was performed in 23.9% (Table 1). Only two (2.3%) thromboembolic events were registered, and both were in the non-residual PH group. Arrhythmic complications were observed in 14.8% of patients (*n* = 13): supraventricular tachycardia in four patients, ventricular extrasystoles in three patients, supraventricular extrasystoles in two patients, ventricular tachycardia in two patients, and atrial fibrillation in two patients as well. Pacemaker implantation was indicated in 9.1% (*n* = 8) of cases. There was no significant difference in the number of complications during follow-up between the residual and non-residual PH groups.

## 4. Discussion

In patients with left-to-right shunt and PH who underwent surgery at a young age, nearly one-third (31%) had residual PH after a follow-up of 9.5 years. These patients showed higher mortality rates in their further life. Patients with more than one shunt were especially prone to developing residual PH. Current publications on residual postoperative PH in congenital systemic-to-pulmonary shunts rarely stratify between different types of CHD, being typically rather old and mostly focusing on PAH or single heart defects (usually ventricular septal defects) [2,14,15]. Therefore, we sought to provide meaningful data for the evaluation of preoperative risks in deciding whether and when to correct congenital systemic-to-pulmonary shunts to prevent the development of residual PH.

We found that PH developed earlier in combined systemic-to-pulmonary shunts compared to complex and simple shunts. We hypothesise that a higher shunt ratio in combined CHD is the most plausible explanation for this finding. As PAH in CHD patients develops due to increased pulmonary blood flow, the combination of pre-tricuspid and post-tricuspid shunts with volume and pressure overload in the pulmonary circulation can result in an early development of PAH [16].

The risk of developing PH after surgical repair of CHD at an older age is reported to be around 15% [17]. According to a recent study that included CHD patients with or without history of surgery, the cumulative incidence of adult PH was 8.3% in patients with systemic-to-pulmonary shunts by the age of 70 [18]. In our study, residual PH was identified in 31% of the patients with CHD after surgical repair, which is probably explained by inclusion selection. In the present study, all patients underwent right heart catheterisation, therefore possibly more severe CHD patients with confirmed PH were included. Additionally, residual PH could have been overestimated, because at follow-up after repair of CHD, we investigated most patients with echocardiography only.

Accurate timing of shunt repair is an ongoing concern in patients with PH and CHD. The main operability criteria include haemodynamic parameters assessed by right heart catheterisation. According to paediatric PH and CHD guidelines, CHD repair should be considered if the indexed pulmonary vascular resistance is <6 Wood units (WU)·m^2^ or pulmonary vascular resistance/systemic vascular resistance is <0.3 at baseline and surgical repair is contraindicated if indexed pulmonary vascular resistance is above this limit [12,19]. The European grown-up congenital heart disease guidelines suggest surgical atrial septal defect or ventricular septal defect closure if pulmonary vascular resistance is <5 WU or Qp/Qs >1.5 [6,20]. Conflicting evidence is published on developing PH after shunt closure and also on closing a shunt in the presence of PH [16,21]. In a recent small-scale study (22 patients), most patients who had developed PAH late after surgical atrial septal or ventricular septal defect closure had pulmonary vascular resistance ≥5 WU (*n* = 18), indexed pulmonary vascular resistance ≥6 WU·m^2^ (*n* = 21), Qp/Qs ≤1.5 (*n* = 11), and pulmonary vascular resistance/systemic vascular resistance ≥0.33 (*n* = 21/22) [16]. Nonetheless, another single-centre retrospective study showed that among 38 patients with surgical repair of VSD and severe PAH (mean pulmonary vascular resistance 7.6 ± 1.8WU), 21% (*n* = 8) died or had developed residual severe PAH after a mean follow-up period of 9 years [21]. No significant baseline haemodynamic differences were found between the groups in terms of worse and favourable outcomes in this study [21]. European guidelines on PH provide even more stringent limits of CHD repair—closure of a systemic-to-pulmonary shunt is recommended if indexed pulmonary vascular resistance <4 WU·m^2^ or pulmonary vascular resistance <2.3 WU and contraindicated if indexed pulmonary vascular resistance >8 WU·m^2^ or pulmonary vascular resistance >4.6 WU. In cases between these ranges (“grey area”), an individual decision after patient evaluation in a tertiary centre is advised [1]. The newest American adult CHD guidelines suggest that pulmonary vascular resistance less than one-third systemic and pulmonary artery systolic pressure lower than 50% systemic are ideal for closing simple shunt lesions [22]. In our study, we found that residual PH after long-term follow-up was associated with higher pulmonary vascular resistance before closure (3.4 vs. 2.2 WU, *p* = 0.035). According to the European Society of Cardiology PH guidelines, our patients with residual PH corresponded to the “grey area” (pulmonary vascular resistance 2.3–4.6 WU) [1]. Still, these recommendations have a poor level of evidence (B [6,12] or C [1,6]) [14,15]. There are no evidence-based recommendations for patients with PAH–CHD considered as borderline for surgery [7]. In these borderline cases, a treat-and-repair strategy could be suggested [19]. Nonetheless, this strategy lacks long-term evidence [7,23].

It is thought that in long-term survival and absence of PH, one of the most important factors is age at surgical repair of CHD [12]. Usually, complete repair of lesions is performed within the first months of life. Direct repair without testing of pulmonary vasculature vasoreactivity is recommended in infants with ventricular septal defect and patent ductus arteriosus until being 6 months old [19]. In our study, 13.6% (*n* = 12) of patients underwent surgical repair prior to being 6 months old. Nonetheless, one recent study showed that PH may still be reversible after repair of CHD with left-to-right shunts in many patients older than 2 years [24]. In our study, there were no age-related differences between the residual and non-residual PH groups. Moreover, some studies report the development of PAH after CHD repair during infancy [17]. Timing of the surgery depends not only on the age of the patient, but also the anatomical location, size of the shunt defect, and presence of a genetic syndrome [25,26]. Therefore, the exact timing of shunt closure is still not precisely defined and we continue to lack factors that could predict reversibility of PH [27]. 

The association between trisomy 21 and residual PH has been noticed [27], and more of our patients with this syndrome had residual PH (22.2% (*n* = 6) vs. 16.4% (*n* = 10)), although statistical significance was not achieved due to low numbers. It was suggested that patients with trisomy 21 are more prone to develop PAH, possibly due to a subtle endothelial dysfunction and upper airway obstruction [26].

All patients with surgically repaired CHD need regular follow-ups for PH in specialised paediatric and later in adult centres, although this is not always successfully accomplished [1,6,12,23]. PH incidence is increasing with time after shunt closure [5,17]. It has been reported that patients with PAH after cardiac defect repair have worse survival than any other clinical PAH–CHD subgroup in adults [17,28]. According to a recent study, CHD adults with PH had more than four times higher all-cause mortality than those without PH [18]. Likewise, we observed higher mortality rates in the residual PH group. In patients with PAH after cardiac defect repair, observed mortality rates at 1, 5, 10, and 20 years after surgery were 2–7%, 17–49%, 35%, and 64% in children, respectively, and at 1, 3, and 5 years were 3%, 6%, and 11% in adults, respectively [4,25,28,29]. Higher mortality rates in patients who received surgical repair of a congenital shunt lesion as children could be due to more complex systemic-to-pulmonary shunts that should be corrected early in childhood.

### Limitations

Our study had several limitations. First, a retrospective, single-centre study design limited the inclusion of higher number of patients or data. Second, the final CHD surgery follow-up group was rather small, and this could have been an obstacle to reach statistically significant differences in our calculations. Additionally, we could not rule out the presumption that excluded patients might have had better outcomes than the follow-up group, although both groups were comparable at baseline. Since a part of the baseline data was collected from old registries covering a long time period (years 1985–2007), some of the height and hemodynamic parameters of right heart catheterisation-related data were incomplete. This may have biased the results of these variables. For the same reason, we only performed logistic regression for sex, age, weight, and pulmonary pressures. Additionally, multivariable analysis was not performed due to the limited number of events. Due to the absence of pulmonary artery wedge pressure and limited pulmonary vascular resistance data during right heart catheterisation, we could not definitely confirm that our final patient group had PAH–CHD according to the current definition of PAH, although it was the most likely diagnosis on the basis of data we had at that time. For the majority of patients, we assessed residual PH after surgery by echocardiography. It could have had an impact on inaccurate evaluation of residual PH. After the surgically repaired CHD, we advised patients to visit the CHD centre regularly; however, not all patients followed this plan. For this reason, we cannot reject a hypothesis that more severe patients visited our healthcare specialist more frequently and stayed in the follow-up. Furthermore, the following data were not purposefully analysed in our study: antenatal use of antithrombotics; the presence of inherited, familial, or acquired thrombophilia; patients’ survival according to the pulmonary arterial pressure assessment method; the probability to develop PH later in life if the patient underwent surgery until 6 months of life; and data of specific drug therapy for PAH.

## 5. Conclusions

PH persisted long-term after the repair of systemic-to-pulmonary shunt in almost one-third of the patients. Residual PH was most often observed in patients with a combined CHD shunt or a higher pulmonary vascular resistance before surgical repair. Residual PH was associated with higher mortality during the follow-up period.

## Figures and Tables

**Table 1:** Baseline characteristics, haemodynamics, and outcomes for all patients stratified according to residual PH after shunt closure.

Variable	No. of Patients	All, *n* = 88	Non-Residual PH after Surgical Correction, *n* = 61 (69.3%)	Residual PH after Surgical Correction, *n* = 27 (30.7%)	*p*-Value
Female sex, *n* (%)	88	44 (50.0)	33 (54.1)	11 (40.7)	0.248
Age at diagnosis (RHC) (years)	88	0.84 (0.61–3.03)	0.84 (0.60–3.60)	0.84 (0.62–2.00)	0.469
Age at surgery (years)	88	1.07 (0.64–3.92)	1.10 (0.64–4.48)	1.04 (0.66–2.30)	0.406
Weight (kg)	72	10.11 ± 5.41	10.45 ± 5.83	9.17 ± 4.00	0.301
Height (cm)	58	83.4 ± 21.4	84.6 ± 22.3	78.9 ± 17.7	0.419
Body surface area (m^2^)	58	0.48 ± 0.19	0.44 ± 0.15	0.49 ± 0.20	0.479
HgB (g/L)	64	117.3 ± 18.9	119.1 ± 18.6	111.7 ± 19.2	0.175
Diagnosis, *n* (%)	88				
ASD		3 (3.4)	3 (4.9)	0 (0)	0.550
APVD		1 (1.1)	1 (1.6)	0 (0)	1.000
PDA		6 (6.8)	5 (8.2)	1 (3.7)	0.662
VSD		26 (29.5)	17 (27.9)	9 (33.3)	0.605
Combined		24 (27.2)	12 (19.6)	12 (44.4)	0.016
TF		3 (3.4)	3 (4.9)	0 (0)	0.550
CAVSD		14 (15.9)	11 (18.0)	3 (11.1)	0.536
TGA		3 (3.4)	3 (4.9)	0 (0)	0.550
TA		1 (1.1)	1 (1.6)	0 (0)	1.000
Other complex defects		7 (8.0)	5 (8.2)	2 (7.4)	1.000
Genetic syndrome, *n* (%)	88	23 (26.1)	16 (26.2)	7 (25.9)	0.976
21st chromosome trisomy		16 (18.2)	10 (16.4)	6 (22.2)	0.626
Other		7 (7.9)	6 (9.8)	1 (3.7)	
RHC before surgery					
PVRi (WU·m^2^)	31	1.45 ± 1.27	2.02 ± 1.88	1.28 ± 1.02	0.336
PVR (WU)	50	3.0 (1.6–4.8)	2.2 (1.0–3.7)	3.4 (2.5–6.5)	0.035
Qp:Qs	45	2.2 (1.4–3.7)	2.3 (1.4–3.9)	1.8 (1.2–2.4)	0.299
TPR (%)	39	34.56 ± 23.09	47.75 ± 16.65	31.16 ± 23.51	0.069
sPAP (mmHg)	88	72.6 ± 26.3	71.3 ± 25.2	75.6 ± 28.9	0.482
dPAP (mmHg)	83	38.78 ± 17.39	37.75 ± 17.46	41.48 ± 17.32	0.385
mPAP (mmHg)	83	51.20 ± 19.84	49.85 ± 19.39	54.74 ± 20.99	0.318
sAoP (mmHg)	88	102.9 ± 21.0	105.6 ± 20.5	96.9 ± 21.3	0.074
dAoP (mmHg)	64	58.1 ± 16.6	57.8 ± 15.7	58.1 ± 19.7	0.784
mAoP (mmHg)	64	74.8 ± 16.0	74.8 ± 14.8	74.7 ± 20.0	0.986
Severity of PH, *n* (%)	88				0.306
Mild		6 (6.8)	6 (9.8)	0 (0)	
Moderate		32 (36.4)	22 (36.1)	10 (37.0)	
Severe		50 (56.8)	33 (54.1)	17 (63.0)	
Duration of follow-up (years)	88	21 (15–24)	21 (16–24)	19 (9–24)	0.187
Complications during follow-up					
Mortality, *n* (%)	88	8 (9.1)	3 (4.9)	5 (18.5)	0.054
NYHA functional class at follow-up, *n* (%)	76				0.173
I and II		49 (64.5)	38 (69.1)	11 (52.4)	
III and IV		27 (35.5)	17 (30.9)	10 (47.6)	
Defect recanalisation, *n* (%)	88	24 (27.3)	16 (26.2)	8 (29.6)	0.775
Repeated surgery, *n* (%)	88	21 (23.9)	14 (23.0)	7 (25.9)	0.763
Thromboembolic event, *n* (%)	88	2 (2.3)	2 (3.3)	0 (0)	1.000
Arrhythmias, *n* (%)	88	13 (14.8)	8 (13.1)	5 (18.5)	0.527
Pacemaker implantation, *n* (%)	88	8 (9.1)	6 (9.8)	2 (7.4)	1.000

Values are number (%), mean (SD) or median (IQR). APVD—anomalous pulmonary venous drainage; ASD—atrial septal defect; CAVSD—complete atrioventricular septal defect; dAoP—diastolic aortic pressure; dPAP—diastolic pulmonary artery pressure; HgB—haemoglobin; mAoP—mean aortic pressure; mPAP—mean pulmonary artery pressure; NYHA—New York Heart Association heart failure classification; PDA—patent ductus arteriosus; PH—pulmonary hypertension; PVR—pulmonary vascular resistance; PVRi—indexed pulmonary vascular resistance; Qp/Qs—ratio of pulmonary to systemic flow; RHC—right heart catheterisation; sAoP—systolic aortic pressure; sPAP—systolic pulmonary artery pressure; TA—truncus arteriosus; TGA—transposition of the great arteries; TF—tetralogy of Fallot; TPR—total peripheral resistance; VSD—ventricular septal defect; WU—Wood units.

**Table 2:** Baseline characteristics and haemodynamics for all patients stratified according to systemic-to-pulmonary shunt type.

Variable	No. of Patients	All, *n* = 88	Simple, *n* = 36 (40.9%)	Combined, *n* = 24 (27.3%)	Complex, *n* = 28 (31.8%)	*p*-Value
Female sex, *n* (%)	88	44 (50.0)	18 (50.0)	9 (37.5)	17 (60.7)	0.248
Age at diagnosis (RHC) (years)	88	0.84 (0.61–3.03)	1.74 (0.69–5.78)	0.65 (0.41–1.40)	0.82 (0.62–3.17)	0.012
Age at surgery (years)	88	1.07 (0.64–3.92)	2.21 (0.76–6.65)	0.74 (0.49–1.68)	0.99 (0.65–3.22)	0.006
Weight (kg)	72	10.11 ± 5.41	11.23 ± 6.20	8.04 ± 4.16	10.29 ± 4.74	0.123
Height (cm)	58	83.4 ± 21.4	89.2 ± 23.8	76.2 ± 18.2	82.8 ± 19.9	0.167
Body surface area (m^2^)	58	0.48 ± 0.19	0.53 ± 0.22	0.41 ± 0.16	0.48 ± 0.18	0.126
HgB (g/L)	64	117.3 ± 18.9	115.5 ± 16.6	112.8 ± 20.0	117.3 ± 18.9	0.152
Genetic syndrome, *n* (%)	88	23 (26.1)	5 (13.9)	7 (29.2)	11 (39.3)	0.067
21st chromosome trisomy		16 (69.6)	2 (40.0)	5 (71.4)	9 (81.8)	0.360
Other		7 (30.4)	3 (60.0)	2 (28.6)	2 (18.2)	
RHC before surgery						
PVRi (WU·m^2^)	31	1.45 ± 1.27	1.62 ± 1.47	1.28 ± 1.05	1.44 ± 1.39	0.817
PVR (WU)	50	3.0 (1.6–4.8)	3.4 (2.0–4.8)	3.0 (1.7–3.5)	2.2 (0.8–6.5)	0.432
Qp:Qs	45	2.2 (1.4–3.7)	2.1 (1.3–2.4)	2.4 (1.4–4.6)	2.7 (1.3–4.6)	0.313
TPR (%)	39	34.56 ± 23.09	25.51 ± 5.57	35.33 ± 22.61	30.56 ± 19.28	0.844
sPAP (mmHg)	88	72.6 ± 26.3	73.5 ± 28.7	72.6 ± 71.4	71.4 ± 24.9	0.952
dPAP (mmHg)	83	38.78 ± 17.39	38.69 ± 17.69	39.55 ± 20.02	38.27 ± 15.14	0.968
mPAP (mmHg)	83	51.20 ± 19.84	51.31 ± 20.76	52.23 ± 21.22	50.19 ± 18.01	0.940
sAoP (mmHg)	88	102.9 ± 21.0	108.3 ± 23.5	99.9 ± 20.9	98.6 ± 16.5	0.131
dAoP (mmHg)	64	58.1 ± 16.6	64.4 ± 15.9	58.1 ± 17.8	49.3 ± 12.7	0.008
mAoP (mmHg)	64	74.8 ± 16.0	81.0 ± 15.3	73.7 ± 17.7	66.9 ± 11.9	0.011
Severity of PH, *n* (%)	88					0.587
Mild		6 (6.8)	4 (11.1)	0 (0)	2 (7.1)	
Moderate		32 (36.4)	13 (36.1)	10 (41.7)	9 (32.1)	
Severe		50 (56.8)	19 (52.8)	14 (58.3)	17 (60.7)	

Values are number (%), mean (SD) or median (IQR). dAoP—diastolic aortic pressure; dPAP—diastolic pulmonary artery pressure; HgB—haemoglobin; mAoP—mean aortic pressure; mPAP—mean pulmonary artery pressure; PH—pulmonary hypertension; PVR—pulmonary vascular resistance; PVRi—indexed pulmonary vascular resistance; Qp/Qs—ratio of pulmonary to systemic flow; RHC—right heart catheterisation; sAoP—systolic aortic pressure; sPAP—systolic pulmonary artery pressure; TPR—total peripheral resistance; WU—Wood units.

**Table 3:** Binary logistic regression analysis of the clinical characteristics and RHC measurements related to residual PH after shunt closure.

Variable (No. of Available Data)	Odds Ratio (OR)	(95% CI)	*p*-Value
Male (*n* = 88)	0.58	(0.23–1.46)	0.250
Age at diagnosis (RHC) (*n* = 88)	0.85	(0.68–1.07)	0.162
Age at surgery (*n* = 78)	0.87	(0.73–1.05)	0.142
Weight (*n* = 72)	0.96	(0.86–1.06)	0.397
sPAP (*n* = 88)	1.01	(0.99–1.02)	0.478
dPAP (*n* = 83)	1.01	(0.99–1.04)	0.381
mPAP (*n* = 83)	1.01	(1.00–1.04)	0.315

RHC—right heart catheterisation; sPAP—systolic pulmonary artery pressure; dPAP—diastolic pulmonary artery pressure; mPAP—mean pulmonary artery pressure.

## Supplementary Materials

The following are available online at https://www.mdpi.com/1010-660X/56/6/297/s1, Figure S1: NYHA functional class at follow-up according to shunt type. Figure S1: NYHA functional class at follow-up according to residual pulmonary hypertension. Table S1: Differences in baseline characteristics between included pulmonary hypertension patients who underwent surgical correction of congenital heart defect with follow-up and patients excluded due to no available follow-up data at our centre.
