# Supplementary material for: Residual Pulmonary Hypertension More than 20 Years after Repair of Shunt Lesions
Source: Medicina (Kaunas). 2020 Jun 16;56(6):297. doi: 10.3390/medicina56060297 (PMC7353861; doi:10.3390/medicina56060297)
Supplement: Supplementary file 1 [file medicina-56-00297-s001.zip › Supplementary Files/Supplementary Table S1.docx]

**Supplementary Table S1:** Differences in baseline characteristics between included pulmonary hypertension patients who underwent surgical correction of congenital heart defect with follow-up and patients excluded due to no available follow-up data at our centre.

| **Variable** | **Follow-up group n=88** | **Without follow-up n=72** | **p value** |
| --- | --- | --- | --- |
| Female sex, n (%) | 44 (50.0%) | 32 (45.1%) | 0.536 |
| Median age at diagnosis (RHC) (years) | 0.84 [0.61 - 3.02] | 1.92 [0.78 - 3.37] | **0.021** |
| Median age at surgery (years) | 1.07 [0.64 - 3.91] | 2.20 [0.86 - 3.60] | 0.074 |
| Height (cm) | 83.41 ± 21.41 | 84.37 ± 20.28 | 0.815 |
| Weight (kg) | 9.00 [6.00 - 13.00] | 9.80 [6.08 - 12.75] | 0.514 |
| Body surface area (m2) | 0.48 ± 0.19 | 0.50 ± 0.21 | 0.663 |
| Shunt type, n (%) |  |  | **0.033** |
| Simple | 36 (40.9) | 44 (61.1) |  |
| Combined | 24 (27.3) | 15 (20.8) |  |
| Complex | 28 (31.8) | 13 (18.1) |  |
| sPAP (mmHg) | 72.61 ± 26.26 | 76.89 ± 24.25 | 0.291 |
| dPAP (mmHg) | 38.78 ± 17.39 | 38.61 ± 18.55 | 0.951 |
| mPAP (mmHg) | 51.20 ± 19.84 | 53.07 ± 19.65 | 0.560 |
| sAP (mmHg) | 102.91 ± 21.03 | 102.89 ± 21.76 | 0.995 |
| dAP (mmHg) | 58.13 ± 16.63 | 57.04 ± 17.76 | 0.746 |
| mAP (mmHg) | 74.75 ± 16.04 | 72.69 ± 19.17 | 0.544 |

Values are number (%), mean (SD) or median (IQR). RHC = right heart catheterization; sPAP = systolic pulmonary artery pressure; dPAP = diastolic pulmonary artery pressure; mPAP = mean pulmonary artery pressure; sAP = systolic aortic pressure; dAP = diastolic aortic pressure; mAP = mean aortic pressure.
