# Supplementary material for: Residual Pulmonary Hypertension More than 20 Years after Repair of Shunt Lesions
Source: Medicina (Kaunas). 2020 Jun 16;56(6):297. doi: 10.3390/medicina56060297 (PMC7353861; doi:10.3390/medicina56060297)
Supplement: Supplementary file 1 [file medicina-56-00297-s001.zip › Supplementary Files/Supplementary Figure legends.docx]

**Supplementary figure legends**

Figure S1: NYHA functional class at follow-up according to shunt type

Figure S2: NYHA functional class at follow-up according to residual pulmonary hypertension

PH - pulmonary hypertension.
