# Supplementary figures and images for: Residual Pulmonary Hypertension More than 20 Years after Repair of Shunt Lesions
Source: Medicina (Kaunas). 2020 Jun 16;56(6):297. doi: 10.3390/medicina56060297 (PMC7353861; doi:10.3390/medicina56060297)

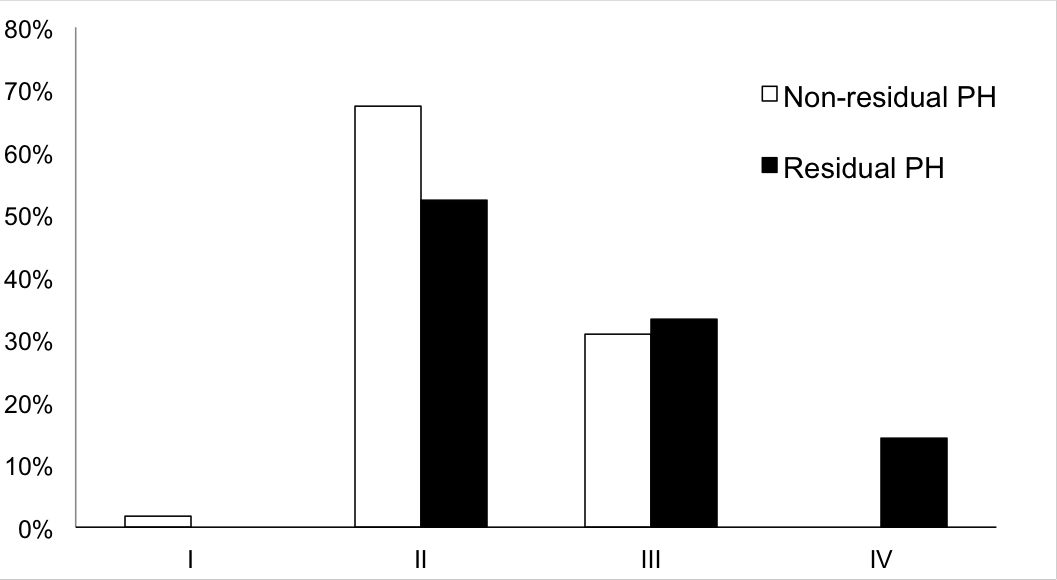

Supplement: Supplementary file 1 [file medicina-56-00297-s001.zip › Supplementary Files/Supplementary Figure S2.tiff]
